# Visceral Obesity and Its Complications: The Role of Bioelectrical Impedance Analysis in Longevity Medicine

**DOI:** 10.3390/metabo16080535

**Published:** 2026-07-29

**Authors:** Mario Mariotti, Valentina Merenda, Francesca Arrigoni, Nadia Tamburlin

**Affiliations:** 1 Executive Committee of Agorà—Italian Society of Aesthetic Medicine, Via San Francesco D’Assisi 4/A, 20122 Milan, Italy; mariotti.mario62@gmail.com (M.M.); dott.nadiatamburlin@gmail.com (N.T.); 2 Agorà—Italian Society of Aesthetic Medicine, Via San Francesco D’Assisi 4/A, 20122 Milan, Italy

**Keywords:** visceral obesity, visceral adipose tissue, bioelectrical impedance analysis, phase angle, inflammaging, sarcopenia, mitochondrial dysfunction, cellular senescence, epigenetic clocks, NAD^+^/sirtuin axis, longevity medicine, cardiometabolic risk, immunometabolism

## Abstract

**Background:** Visceral obesity is increasingly recognised not as a simple excess of adipose tissue, but as a systemic pathological condition characterised by profound metabolic, endocrine, and immune dysregulation. Visceral adipose tissue (VAT) operates as an autonomous neuro-immune-endocrine organ whose dysfunctional expansion drives insulin resistance, atherogenesis, and accelerated cellular ageing through mechanisms converging on chronic low-grade sterile inflammation, referred to as inflammaging. **Objectives:** This narrative review integrates evidence across four domains: (1) the multi-system clinical complications of visceral obesity and the methodological controversies surrounding its measurement; (2) the cellular heterogeneity, immunometabolic reprogramming, and molecular mechanisms through which excess VAT accelerates biological ageing, with a focus on genomic instability, mitochondrial dysfunction, the NAD^+^/sirtuin regulatory axis, cellular senescence, and inter-organ communication; (3) the role of bioelectrical impedance analysis (BIA)—particularly phase angle—as a non-invasive functional biomarker of biological age and longevity, positioned critically against alternative assessment methods; and (4) current knowledge gaps and priorities for future research. **Methods:** A narrative review of PubMed/MEDLINE, Google Scholar, and the Cochrane Library was conducted using MeSH terms and free-text keywords including visceral obesity, bioelectrical impedance analysis, phase angle, sarcopenia, inflammaging, mitochondrial dysfunction, cellular senescence, epigenetic clocks, NAD^+^, sirtuin, and longevity, supplemented by citation-tracking of retrieved reviews. English-language articles published up to April 2025 were considered, prioritising systematic reviews, meta-analyses, and prospective cohort studies; formal risk-of-bias tools and quantitative synthesis were not applied, consistent with a narrative review design. **Results and Discussion:** BIA-derived phase angle constitutes a macroscopic electrobiological correlate of inflammaging: low phase angle values in visceral obese subjects overlap with those of frail elderly individuals, reflecting impaired membrane integrity, loss of active cell mass, and altered ICW/ECW balance. However, this evidence base remains largely cross-sectional and correlative; the directionality and population-specific calibration of BIA-derived indices constitute the principal unresolved methodological questions. Integration with epigenetic clocks, circulating NAD^+^ levels, and gut microbiome indices offers a framework for dynamic biological age assessment, though prospective interventional validation is still lacking. Sarcopenic obesity, evaluated through EWGSOP2 combined with BIA-derived skeletal muscle mass index and handgrip dynamometry, represents a critical comorbidity demanding integrated therapeutic targeting. **Conclusions:** BIA provides a quantitative, accessible correlate for translating cellular metabolic health into clinically actionable parameters, complementary to rather than a replacement for anthropometric and imaging-based methods. Optimising phase angle and reducing VAT through anti-inflammatory nutrition, exercise, and nutraceutical strategies targeting the NAD^+^/sirtuin and mTOR/AMPK axes constitutes a measurable objective for the promotion of healthy longevity, contingent on the longitudinal, mechanistic studies identified as priorities in this review.

## 1. Introduction

The global epidemic of obesity has progressed beyond a nutritional disorder into one of the most consequential challenges of contemporary medicine. According to the GBD 2015 Obesity Collaborators, overweight and obesity now affect more than two billion individuals across 195 countries, with age-standardised prevalence having doubled in most high-income nations since 1980 [1,2]. The traditional clinical approach has relied on body mass index (BMI)—introduced by Quetelet in the nineteenth century—as the primary diagnostic criterion. However, BMI conflates fat mass with lean mass, disregards regional fat distribution, and fails to identify individuals with high cardiometabolic risk who present with a normal-weight phenotype [3,4]. This limitation has prompted the development of staging frameworks that move beyond simple anthropometry toward functional and complication-based obesity classification, though consensus on which framework should guide clinical decision-making remains unsettled [5,6,7].

The pivotal conceptual shift came with recognition that it is the anatomical distribution and metabolic quality of adipose tissue, rather than its total quantity, that determines biological risk. Visceral adipose tissue (VAT)—accumulating within the abdominal cavity and surrounding the visceral organs—behaves in a fundamentally different manner from subcutaneous fat. Rather than functioning as an inert energy reservoir, VAT operates as a metabolically hyperactive neuro-immune-endocrine organ, continuously synthesising and releasing bioactive molecules that modulate insulin signalling, vascular function, immune activation, and the molecular machinery of biological ageing [8,9,10,11].

This review is organised around a specific thesis: visceral obesity and biological ageing share a common mechanistic infrastructure. The chronic low-grade sterile inflammation, oxidative stress, mitochondrial dysfunction, and genomic instability that characterise the visceral obese phenotype are precisely the cellular processes that define biological ageing. A landmark multicohort study demonstrated that obesity is associated with significantly elevated risk for 45 of 83 hallmark-related diseases of ageing, providing one of the most comprehensive epidemiological correlations of this mechanistic convergence to date; however, association at the epidemiological level does not, by itself, establish the causal and temporal sequence proposed here, and this distinction is revisited critically throughout the review [12,13].

**Figure 1:**
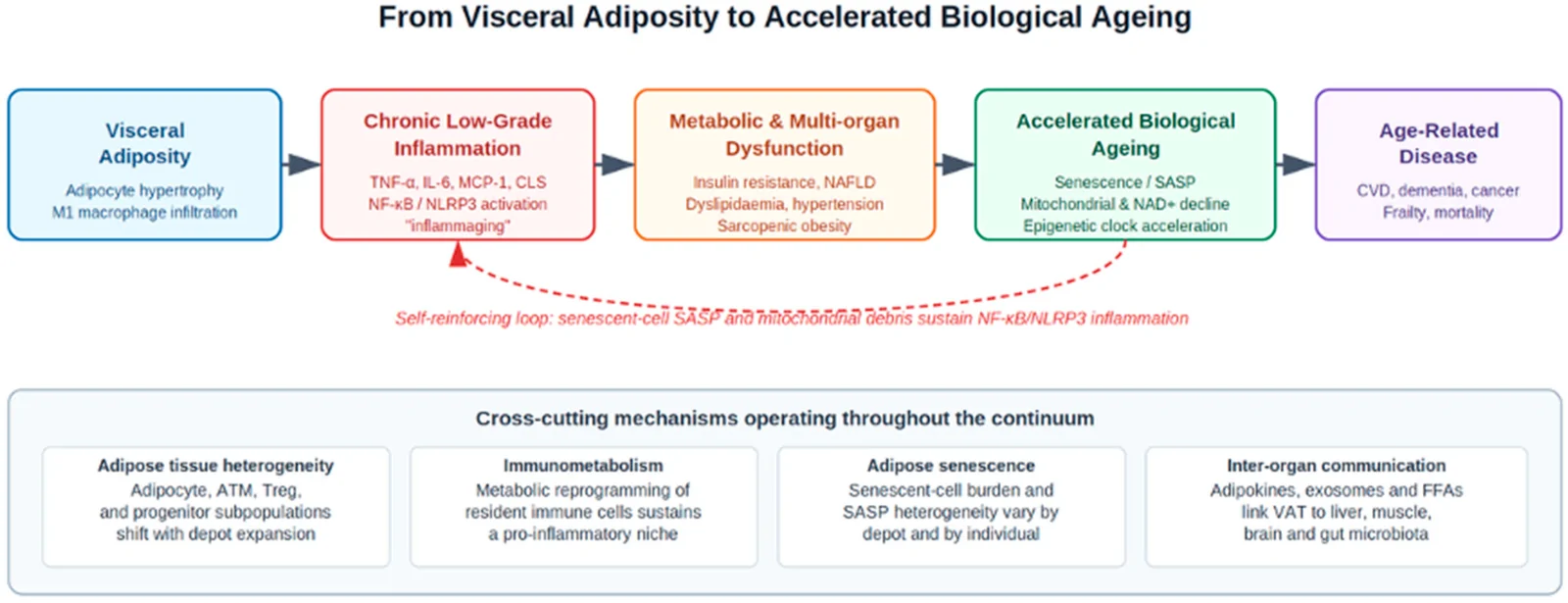
Mechanistic progression from visceral adiposity to age-related disease, highlighting cross-cutting mechanisms discussed critically throughout Section 3 and Section 4.

Against this background, bioelectrical impedance analysis (BIA) acquires a significance that extends beyond standard body composition measurement. Phase angle (PhA)—the ratio between cellular reactance and resistance, reflecting membrane integrity and active cellular mass—has been proposed as a non-invasive macroscopic readout of cellular biological age: a parameter that declines in parallel in visceral obesity and chronological ageing, and whose normalisation through targeted intervention constitutes a candidate longevity objective. This review evaluates that proposal critically, alongside competing and complementary assessment methods, rather than treating it as an established clinical standard [14,15,16]. The overall mechanistic framework is summarised in Figure 1.

## 2. Methods

### 2.1. Search Strategy and Information Sources

A narrative review of the literature was conducted using PubMed/MEDLINE as the primary bibliographic database, supplemented by targeted searches of Google Scholar and the Cochrane Library to identify systematic reviews and meta-analyses not indexed with the specific MeSH terms used in the primary search. The following MeSH terms and free-text keywords were combined using Boolean operators (AND/OR): “visceral obesity”, “visceral adipose tissue”, “adipokines”, “bioelectrical impedance analysis”, “phase angle”, “BIVA”, “body composition”, “inflammaging”, “cellular senescence”, “SASP”, “sarcopenia”, “EWGSOP2”, “mitochondrial dysfunction”, “NAD+ metabolism”, “sirtuin”, “telomere length”, “epigenetic clock”, “longevity”, “cardiometabolic risk”, and “insulin resistance”. Reference lists of retrieved reviews were hand-searched for additional relevant primary studies (“snowball” or citation-tracking approach).

### 2.2. Eligibility Criteria

English-language articles published between January 2000 and April 2025 were considered. Inclusion criteria prioritised, in descending order of preference: (1) systematic reviews and meta-analyses; (2) prospective cohort studies; (3) other original human studies (cross-sectional or case–control) providing data not otherwise available; and (4) mechanistic studies in cellular or animal models, included selectively when they provided molecular insight not obtainable from human data. Case reports, conference abstracts without a full peer-reviewed publication, non-English-language articles, and studies not directly addressing visceral adiposity, BIA/phase angle, or the specific ageing mechanisms under review were excluded. Where multiple studies addressed the same narrow question, more recent or larger studies, and systematic reviews over individual primary studies, were preferentially retained to keep the reference list focused.

### 2.3. Study Selection and Evidence Prioritisation

Titles and abstracts identified through the search strategy were screened by the corresponding authors for topical relevance to the four domains outlined in the Objectives; full texts were retrieved for records passing this initial screen, and a final selection was made based on methodological quality (study design, sample size, and, for human studies, adjustment for relevant confounders) and direct relevance to the review’s thesis. This screening was conducted by the author team rather than through a pre-registered, dual-reviewer systematic process; consequently, no PRISMA flow diagram, formal inter-reviewer agreement statistic, or quantitative risk-of-bias tool (e.g., ROBIS, AMSTAR-2) was applied. As a narrative rather than systematic review, no meta-analytic quantitative synthesis was performed. This lack of a formal, reproducible selection protocol is an acknowledged methodological limitation of the present work and is revisited explicitly in Section 7, where a fully systematic review of the BIA-longevity literature is identified as a priority for future work.

## 3. Visceral Adipose Tissue: Cellular Heterogeneity, Immunometabolism, and Multi-System Complications

### 3.1. VAT as a Neuro-Immune-Endocrine Organ: Cellular Heterogeneity and Adipokine Dysregulation

The pathological behaviour of expanded VAT stems from a fundamental reprogramming of adipocyte secretory function, but adipose tissue is not a homogeneous effector: mature adipocytes coexist with preadipocytes, adipose-derived stem/stromal cells, endothelial cells, fibroblasts, and a heterogeneous immune infiltrate whose relative proportions shift substantially with depot expansion. This cellular heterogeneity is increasingly recognised as a determinant of VAT pathogenicity in its own right, rather than a background detail: single-cell transcriptomic studies have begun to resolve distinct adipocyte and progenitor subpopulations with divergent inflammatory and fibrotic potential, though this literature is still emerging and has not yet been translated into clinical stratification tools.

Under physiological conditions, adipose tissue contributes to systemic homeostasis through the secretion of adiponectin—an insulin-sensitising, anti-atherogenic, and anti-inflammatory adipokine—and leptin, which regulates satiety via hypothalamic signalling. Under conditions of chronic energy surplus, visceral adipocytes undergo hypertrophy and functional reprogramming: adiponectin secretion is progressively suppressed, while the production of pro-inflammatory adipokines—including tumour necrosis factor-alpha (TNF-α), interleukin-6 (IL-6), monocyte chemoattractant protein-1 (MCP-1), resistin, and visfatin—is markedly upregulated [10,11,17].

The mechanistic consequences of this shift are systemic. TNF-α and IL-6 activate the NF-κB and JNK intracellular signalling cascades in hepatic, muscular, and vascular tissues, directly impairing insulin receptor substrate-1 (IRS-1) serine phosphorylation and downstream PI3K/AKT signalling, establishing the molecular basis of peripheral insulin resistance. IL-6, released by VAT in quantities proportional to depot size, drives hepatic CRP synthesis. Resistin and visfatin exacerbate endothelial dysfunction through complementary receptor-mediated pathways. The net result is a state of persistent, low-grade systemic inflammatory activation that is metabolically destabilising and a primary driver of accelerated cellular ageing [10,11,17,18,19].

This inflammatory state is amplified by the infiltration of macrophages into the hypertrophied visceral depot, where they form characteristic crown-like structures (CLS) around necrotic adipocytes. Immunometabolism—the reciprocal relationship between the metabolic state of immune cells and their functional polarisation—offers a more mechanistic framework than the traditional M1/M2 dichotomy for understanding this process: adipose tissue macrophages in the obese depot shift toward glycolytic, pro-inflammatory metabolic programmes, while lipid handling and hypoxia within the expanding depot further reinforce this polarisation, producing additional TNF-α, IL-1β, and reactive nitrogen species. The clinical correlates are measurable: hs-CRP values ≥ 3 mg/L, elevated fibrinogen and ferritin, and reduced serum albumin collectively define the inflammatory signature of visceral obesity. The Dietary Inflammatory Index (E-DII) provides a validated tool for estimating the dietary contribution to this systemic inflammatory load, though it remains a surrogate rather than a direct cellular measure [19,20].

Despite this mechanistic detail, it should be noted that much of the human evidence linking specific adipokine profiles to clinical outcomes remains correlative and cross-sectional; causal inference is drawn largely from cellular and animal models, and the relative contribution of adipocyte-derived versus immune-cell-derived mediators in human VAT dysfunction is not fully resolved.

### 3.2. Cardiometabolic, Hepatic, and Endocrine Complications

The cardiometabolic consequences of VAT dysfunction are mediated principally through the portal circulation. VAT-derived free fatty acids (FFAs), released in excess due to the high lipolytic activity of visceral adipocytes, reach the liver in concentrations sufficient to stimulate de novo gluconeogenesis, impair hepatic insulin clearance, and drive de novo lipogenesis. The resulting dyslipidaemia is characterised by elevated triglycerides, small-dense LDL particles, and reduced HDL cholesterol. Concurrent hepatic lipid accumulation underlies non-alcoholic fatty liver disease (NAFLD), which may progress to steatohepatitis (NASH) and fibrosis in susceptible individuals [18,21,22].

Beyond dyslipidaemia, visceral obesity is an independent predictor of hypertension—mediated through sympathetic nervous system activation, renin–angiotensin–aldosterone system upregulation, and endothelial nitric oxide synthase inhibition by asymmetric dimethylarginine (ADMA)—and of type 2 diabetes mellitus through combined insulin resistance and progressive beta-cell exhaustion. Kuk et al. demonstrated in a prospective cohort that visceral fat area predicted all-cause mortality in men independently of total adiposity and BMI, underscoring the primacy of fat distribution over fat quantity in risk stratification [17,18,23].

Polycystic ovary syndrome (PCOS) represents a relevant endocrine complication: hyperinsulinaemia directly stimulates ovarian theca cell androgen production via insulin receptor-mediated LH sensitisation, disrupts follicular maturation, and inhibits ovulation independently of BMI. Obstructive sleep apnoea syndrome (OSAS) is similarly driven by VAT-mediated endothelial dysfunction and HIF-1α upregulation, contributing to upper airway collapsibility and nocturnal desaturation, which in turn amplify insulin resistance and systemic inflammation [10,22].

### 3.3. Posturomotor and Musculoskeletal Implications

Excess abdominal adiposity anteriorly displaces the centre of gravity, imposing compressive loading on the lumbar spine and generating compensatory adaptations including hyperlordosis, increased thoracic kyphosis, and progressive myofascial tension along the posterior kinetic chain. Gait analysis studies confirm that a higher BMI correlates more strongly with altered locomotor parameters—reduced walking speed, asymmetric ground reaction forces, altered swing-to-stance ratio—than the relative proportion of fat mass, indicating that absolute mechanical load, and not fat distribution per se, governs locomotor impairment in this domain—a useful reminder that not every complication of obesity is mediated through the VAT-inflammaging axis emphasised elsewhere in this review [24,25].

A meta-analysis of 33 studies demonstrated a significantly higher prevalence of chronic low back pain in obese subjects, operating through at least three parallel mechanisms: increased mechanical compression of intervertebral discs and vertebral endplates; VAT-derived systemic inflammation contributing to discogenic sensitisation and nociceptive modulation; and accelerated degenerative disc changes driven by metabolic and oxidative stress. In elderly subjects, the mechanical and inflammatory burden of visceral obesity compounds with sarcopenic muscle loss—whose pathogenesis and relationship to fat mass are addressed mechanistically in Section 5.4—to produce a phenotype characterised by impaired balance, recurrent falls, and accelerated functional decline [24,26,27].

### 3.4. Neurological and Oncological Associations

Increased abdominal fat mass is associated with accelerated brain ageing and Alzheimer’s-like cerebral atrophy, with effects more pronounced in males. The mechanisms are coherent: insulin resistance impairs amyloid-beta clearance through competition between insulin-degrading enzyme (IDE) and amyloid-beta for substrate binding; hyperphosphorylation of tau is promoted by GSK-3β, constitutively activated in insulin-resistant states; and VAT-derived adipokines—particularly leptin resistance and elevated TNF-α—activate microglial neuroinflammation via NF-κB. This adipo-cerebral axis is one of several examples of inter-organ communication discussed synthetically in Section 4.5 [8,28,29].

The oncological implications operate through three convergent molecular pathways: hyperinsulinaemia and elevated IGF-1 activate the PI3K/AKT/mTOR proliferative cascade, suppressing apoptosis and promoting cell cycle progression; aromatase overexpression in hypertrophied adipocytes drives local oestrogen excess, with relevance particularly for breast, endometrial, and ovarian cancers; and chronic NF-κB and COX-2 activation generates a pro-mutagenic microenvironment. Epidemiological data confirm an association between increasing BMI and the risk of at least 22 distinct cancers, a body of evidence that is robust but, again, anchored to BMI rather than to direct measures of visceral adiposity—a methodological limitation discussed in Section 3.5 [30].

### 3.5. Methodological Controversies in the Assessment of Visceral Adiposity

The complications described above have been established using a heterogeneous mix of anthropometric, imaging, and bioimpedance methods, and this heterogeneity is itself a limitation of the current evidence base rather than a peripheral technical detail. BMI remains the most widely used index for historical and practical reasons, but it cannot distinguish fat from lean mass or subcutaneous from visceral depots [3,4]. Waist circumference and the waist-to-hip ratio improve on BMI by capturing central adiposity, yet both remain surrogate measures that do not directly quantify visceral fat area and are influenced by measurement technique, posture, and observer variability.

Computed tomography (CT) and magnetic resonance imaging (MRI) are considered reference-standard methods for direct visceral fat area quantification, but their cost, radiation exposure (for CT), and limited accessibility preclude routine clinical use, restricting their role to research settings and select high-risk populations. Dual-energy X-ray absorptiometry (DXA) offers a reasonable compromise for total and regional fat mass with modest radiation exposure, but its visceral fat estimates are derived from validated equations rather than direct visualisation and require population-specific calibration.

**Figure 2:**
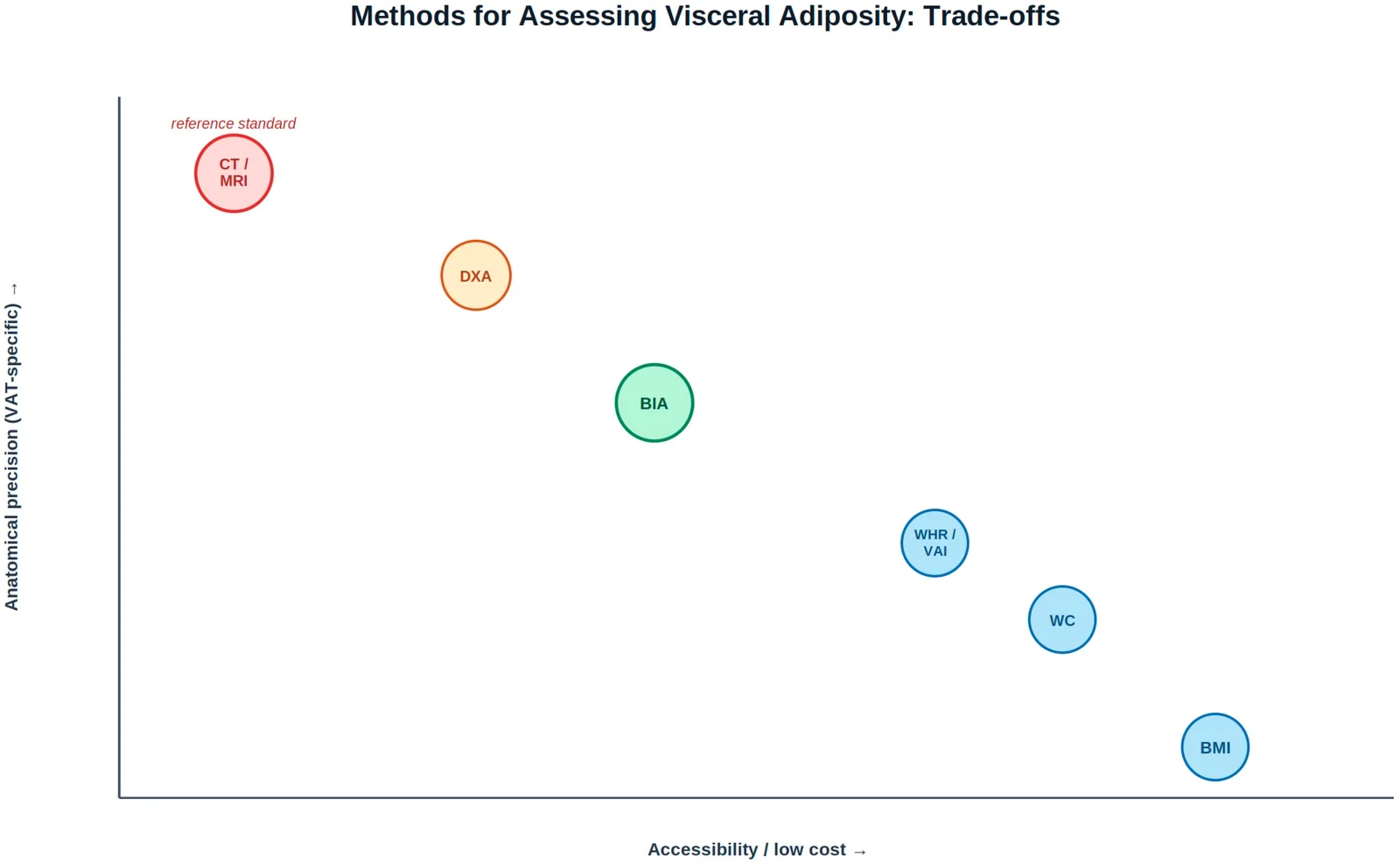
Methods for assessing visceral adiposity: relative accessibility, cost, and directness (see Section 3.5 and Table 2).

Bioelectrical impedance analysis (BIA), the focus of this review from Section 5 onward, is inexpensive, portable, and radiation-free, but visceral fat area and phase angle estimates from BIA are themselves population-, ethnicity-, and equation-specific, and direct comparative studies between BIA-derived and imaging-derived indices show only moderate agreement in some populations [31]. Comparative work examining bioelectrical versus anthropometric indices in relation to hypertension risk illustrates this point: agreement between methods is method- and outcome-dependent rather than uniform, and no single index currently outperforms the others across all clinical contexts [31].The BIA-specific parameters are detailed in Table 1. Table 2 summeries the comparative applicability, strengths, and limitations of these methods; Figure 2 presents this comparison graphically. A central unresolved question for the field—and a stated priority for future research in this review—is whether BIA-derived indices, once properly calibrated and validated against imaging in longitudinal cohorts, can substitute for or must remain adjunctive to CT/MRI-based reference standards in specific clinical decisions.

## 4. Visceral Obesity as an Accelerator of Biological Ageing: Molecular Mechanisms

### 4.1. The Hallmarks of Ageing and Their Activation by VAT

The biological ageing process is governed by nine interacting cellular and molecular mechanisms: genomic instability, telomere attrition, epigenetic alterations, loss of proteostasis, deregulated nutrient sensing, mitochondrial dysfunction, cellular senescence, stem cell exhaustion, and altered intercellular communication. These hallmarks form a network of mutually reinforcing feedback loops in which dysfunction in one amplifies the others, generating a self-accelerating trajectory towards frailty and age-related disease. Visceral obesity does not engage with a subset of these hallmarks selectively—epidemiological and mechanistic data together suggest it engages all nine to varying degrees, positioning excess VAT as one of the most clinically prevalent systemic accelerators of biological ageing identified to date. It should be emphasised, however, that the strength of evidence varies considerably across these nine hallmarks: genomic instability, mitochondrial dysfunction, and cellular senescence are supported by convergent human and mechanistic data, whereas the links to stem cell exhaustion and altered intercellular communication remain comparatively less direct in human VAT and warrant the dedicated mechanistic studies called for throughout this section [12,13].

### 4.2. Genomic Instability, Telomere Attrition, and Epigenetic Clock Acceleration

VAT-driven overproduction of reactive oxygen species (ROS), combined with the direct genotoxic action of pro-inflammatory cytokines, inflicts chronic DNA damage: double-strand breaks accumulate faster than nucleotide excision repair (NER) and base excision repair (BER) mechanisms can resolve them, leading to persistent DNA damage response (DDR) activation. DDR signalling through ATM/ATR kinases activates the cGAS-STING innate immune pathway and the NF-κB transcription factor, establishing a positive feedback loop in which DNA damage sustains inflammation and inflammation impairs DNA repair [12,32].

Telomere attrition is accelerated by the same oxidative and inflammatory conditions, since telomeric guanine-rich sequences are preferentially susceptible to oxidative damage and inefficiently repaired. Dysfunctional telomeres activate the NLRP3 inflammasome and increase mitochondrial superoxide production through retrograde signalling, creating a bidirectional amplification loop between telomeric dysfunction and systemic inflammation. These changes are quantifiable through DNA methylation-based epigenetic clocks—Horvath’s pan-tissue clock, Hannum’s blood clock, and PhenoAge—which consistently reveal epigenetic age acceleration in obese subjects correlating more strongly with visceral fat area than with BMI or total fat mass, although the number of studies pairing epigenetic clocks with directly quantified visceral fat area (rather than BMI) remains small [12,32,33].

### 4.3. Mitochondrial Dysfunction and the NAD^+^/Sirtuin Regulatory Axis

Mitochondria occupy the functional centre of biological ageing. The electron transport chain drives ATP synthesis via oxidative phosphorylation while generating a controlled ROS flux. The endogenous antioxidant system—principally superoxide dismutase (SOD1/SOD2) and catalase—neutralises these ROS under physiological conditions. However, mitochondrial antioxidant capacity declines at approximately 10% per decade from the third decade of life, a rate substantially accelerated under obesity-driven metabolic stress, insulin resistance, and chronic cytokine exposure [34].

When ROS generation overwhelms neutralisation capacity, oxidative stress triggers cardiolipin peroxidation in the inner membrane, mtDNA mutation accumulation, and impaired ATP synthesis. Damaged mitochondria that escape mitophagy release mtDNA and cardiolipin into the cytosol, activating cGAS-STING and the NLRP3 inflammasome, perpetuating the inflammatory state. This cellular waste accumulation—described as “garb-aging”—creates a self-reinforcing cycle of mitochondrial dysfunction and chronic inflammation. Notably, weight loss alone does not appear sufficient to reverse this state: remission of obesity has been shown not to fully restore mitochondrial homeostasis in visceral adipose tissue, indicating a degree of persistent, possibly hysteretic, cellular damage that current therapeutic paradigms—centred on weight reduction—may not adequately address; this finding is revisited in the Discussion as a central argument for expanding treatment endpoints beyond weight [34].

Sirtuins (SIRT1-SIRT7) are NAD^+^-dependent protein deacylases representing a key molecular interface between energy metabolism and longevity. SIRT3 and SIRT4, the principal mitochondrial sirtuins, activate key oxidative metabolism enzymes and stimulate mitophagy through PINK1/Parkin pathway regulation. SIRT1 deacetylates PGC-1α, a master regulator of mitochondrial biogenesis, promoting replacement of dysfunctional mitochondria. This regulatory programme requires NAD^+^ as an obligate cofactor—and NAD^+^ levels decline progressively with both chronological ageing and visceral obesity, as chronic NF-κB activation consumes NAD^+^ through PARP-mediated DNA repair and NAMPT expression is suppressed [34,35].

The mTOR/AMPK axis represents the reciprocal counterpart of the sirtuin system. AMPK, activated by a falling ATP/AMP ratio, promotes mitophagy, inhibits mTORC1-driven anabolism, and stimulates NAD^+^ biosynthesis, thereby activating sirtuins. Chronic overnutrition and VAT expansion constitutively hyperactivate mTORC1 and suppress AMPK, locking cells in an anabolic state that impedes mitochondrial quality control, accumulates cellular damage, and promotes the senescence transition. Caloric restriction and aerobic exercise reverse this balance through AMPK activation, generating the molecular conditions for longevity, though the durability of these effects and the optimal dose and duration of intervention remain active areas of investigation [35].

### 4.4. Cellular Senescence, the SASP, and Inflammaging

Cellular senescence—the stable, irreversible arrest of cell cycle progression triggered by DNA damage beyond repair capacity, telomere dysfunction, or proteotoxic stress—accumulates when senescent cells exceed the clearance capacity of NK cells and cytotoxic T lymphocytes, a condition that worsens with both chronological ageing and visceral obesity, as chronic inflammation progressively impairs immune surveillance. Senescent cell burden in human VAT is heterogeneous across depots and individuals, and the field has not yet fully resolved whether senescent adipocytes are a primary driver of the surrounding pathology or, in part, a bystander consequence of the inflammatory microenvironment described in Section 3.1—a distinction with direct implications for the rationale of senolytic therapeutic strategies, which lie outside the scope of the interventions reviewed in Section 6 [13,19].

Accumulated senescent cells exert deleterious effects through the senescence-associated secretory phenotype (SASP): the constitutive secretion of pro-inflammatory cytokines (IL-6, IL-1β, IL-8), chemokines (CXCL1, CXCL10, MCP-1), growth factors (HGF, VEGF, TGF-β), and matrix metalloproteinases (MMP-1, MMP-3, MMP-9). The SASP remodels the local extracellular matrix, recruits myeloid immune cells, and induces paracrine senescence in adjacent cells through “senescence contagion” mediated by ROS and gap junction signalling [12,13,19].

The systemic expression of this process is inflammaging: the chronic, sterile, low-grade inflammatory state characterised by persistently elevated IL-6, TNF-α, IL-1β, and hs-CRP that defines the ageing phenotype, and whose upstream adipokine drivers were introduced in Section 3.1. Inflammaging feeds back into each remaining hallmark of ageing: it accelerates telomere attrition through telomerase inhibition by TNF-α; impairs proteostasis by blocking proteasomal degradation and autophagy initiation; disrupts stem cell niche homeostasis through TGF-β and Wnt pathway interference; and uncouples mitochondrial oxidative phosphorylation through NF-κB-mediated suppression of SIRT3 and PGC-1α [12,13,19].

### 4.5. Inter-Organ Communication: Synthesising the Adipo-Centric Axes of Ageing

The complications and mechanisms described in Section 3 and Section 4 are frequently presented, in the existing literature, as organ-specific narratives—hepatic, cerebral, muscular, intestinal—that risk obscuring their shared origin in VAT-derived signalling. Framed instead as inter-organ communication, a coherent picture emerges. The adipo-hepatic axis, mediated by portal FFA flux and hepatokine signalling, links VAT expansion directly to NAFLD/NASH progression. The adipo-cerebral axis, mediated by insulin resistance, leptin signalling, and circulating inflammatory mediators crossing an increasingly permeable blood–brain barrier, links VAT to accelerated brain ageing and neurodegenerative risk. The adipo-gut axis operates bidirectionally: intestinal dysbiosis and barrier permeability associated with visceral obesity allow lipopolysaccharide (LPS) and other dysbiotic metabolites to enter systemic circulation and trigger constitutive TLR4-mediated immune activation, sustaining the inflammaging loop independently of adipose tissue signals, while VAT-derived inflammatory mediators reciprocally shape gut barrier integrity and microbial composition. Multi-organ metabolomic approaches that integrate signals across these axes into composite biological age estimates represent an emerging and still largely exploratory methodology, illustrating both the promise and the current immaturity of an integrated, systems-level model of VAT-driven ageing [8,22,36].

## 5. Bioelectrical Impedance Analysis: Principles, Parameters, and the Biology of Phase Angle

### 5.1. Biophysical Foundations and Measurement Standards

Bioelectrical impedance analysis is founded on the differential electrical conductivity of biological tissues: lean tissues—muscle, blood, cerebrospinal fluid—are rich in water and electrolytes and conduct an alternating current (AC) efficiently, while adipose tissue, bone, and air-filled structures are poor conductors. A low-intensity AC of approximately 800 μA is delivered through the body via skin-contact electrodes. The total impedance (Z) is decomposed into resistance (R), representing the opposition of the electrolytic aqueous phase and inversely proportional to fat-free mass (FFM), and reactance (Xᴄ), representing the capacitive opposition generated by cell membranes acting as biological capacitors. Their relationship is described by Z^2^ = R^2^ + Xᴄ^2^ [14,15].

Phase angle (PhA = arctan[Xᴄ/R] × 180/π) integrates both components into a single parameter reflecting the ratio between intracellular and extracellular fluid volumes and the functional integrity of cell membranes. Higher PhA values indicate greater cellular mass, structurally intact membranes, and favourable intracellular hydration. Reference values in healthy adults range from 5° to 8°, with optimal values at 6–7°; values below 4° indicate cellular or nutritional compromise; values below 2° reflect severe depletion. Modern multi-frequency, 8-electrode systems enable segmental body composition analysis and generate visceral fat area (VFA) estimates through population-specific predictive models, whose accuracy remains dependent on the ethnic and clinical calibration cohort used to derive them [14,15,16].

Measurement validity requires standardised pre-measurement conditions: fasting for at least 4 h, abstinence from exercise for 12 h, from alcohol for 48 h, and from diuretics for 7 days; room temperature at 20–24 °C at approximately 50% relative humidity. Multi-frequency systems are essential for the detection of ICW/ECW compartmental shifts central to the metabolic assessment of visceral obesity and ageing; mono-frequency devices cannot perform this differentiation.

### 5.2. Established Clinical Applications of Phase Angle

Before considering its proposed role in visceral obesity and ageing, it is important to state plainly what phase angle is already validated to do, since the strength of evidence differs substantially between this established use and the more exploratory applications discussed in Section 5.3. As a prognostic marker of nutritional and functional status, phase angle has been validated in large, disease-specific cohorts across oncology, nephrology, hepatology, cardiology, and critical care: lower phase angle consistently and independently predicts adverse outcomes (mortality, complications, length of stay) in malignancy, chronic kidney disease (including dialysis populations), liver cirrhosis, heart failure, and critical illness, generally outperforming or complementing conventional anthropometric parameters such as BMI or albumin in these specific clinical contexts. In the general population, phase angle correlates inversely with hs-CRP, IL-6, and TNF-α, and positively with handgrip strength, Short Physical Performance Battery score, and quality-of-life measures—associations that are well replicated across multiple independent cohorts [14,16,20].

These uses share three features that justify calling them “established”: validation in disease-specific populations with hard clinical endpoints (mortality, hospital readmission), replication across multiple independent cohorts, and incorporation into nutritional-status and frailty-assessment protocols already used in some clinical settings (e.g., oncology and dialysis nutrition assessment). None of these established applications, however, was designed or validated to test phase angle specifically as a marker of visceral obesity or of biological ageing as a general construct; extending its use to those domains, discussed next, rests on a different and considerably thinner evidence base.

### 5.3. Phase Angle as a Candidate Biomarker of Inflammaging and Biological Ageing in Visceral Obesity: An Exploratory Extension

The application of phase angle specifically to visceral obesity and to biological ageing—as distinct from the disease-specific prognostic uses above—is comparatively recent and should be regarded as exploratory rather than established. The central observation motivating this extension is an electrobiological similarity between the BIA vector pattern of visceral obese subjects and that of frail elderly individuals: low phase angle, reduced reactance Xᴄ, elevated ECW/TBW ratio, and a vector displaced towards the lower-right quadrant of the BIVA plot, a pattern some authors have labelled an “inflammaging BIA signature”. This pattern is proposed—but not yet mechanistically confirmed—to reflect the cellular pathology shared by biological ageing and visceral obesity: impaired membrane polarisation, reduced active cell mass, and altered fluid compartmentalisation resulting from SASP-driven tissue remodelling and chronic inflammatory catabolism. It should be stressed plainly that this remains a pattern-recognition finding derived largely from cross-sectional comparisons rather than a validated biomarker pathway: no study to date has followed individuals longitudinally through the transition into visceral obesity or frailty with paired, repeated BIA measurements to establish the temporal and causal relationship this signature implies [15,16,32].

Consistent with this exploratory status, subjects with visceral obesity display significantly lower PhA values than age- and sex-matched normal-weight controls even in the absence of overt metabolic syndrome criteria, a finding compatible with (but not proof of) PhA as a sensitive biomarker for subclinical metabolic and cellular dysfunction in the “metabolically obese, normal-weight” phenotype. This specific application—unlike the disease-specific prognostic uses in Section 5.2—has not been validated in dedicated prospective cohorts designed to test it, and should be communicated to clinicians and patients as a research-stage hypothesis rather than a validated diagnostic tool [14,16,20,37].

### 5.4. BIA in the Diagnosis of Sarcopenic Obesity: Integration with EWGSOP2 Criteria

Sarcopenia—the progressive, generalised loss of skeletal muscle mass and function (ICD-10-CM M62.84)—represents a critical comorbidity of visceral obesity, and its pathogenesis is best understood as multifactorial rather than reducible to a single adipokine pathway: VAT-derived TNF-α suppresses myoblast differentiation through NF-κB-mediated inhibition of MyoD transcription factor; IL-6 activates the ubiquitin-proteasome pathway and muscle protein catabolism; myostatin, paradoxically upregulated in obese adipose tissue, inhibits muscle stem cell activation; and, in parallel, sex hormone status independently modulates both fat distribution and muscle mass, adding a further layer of individual variability that a purely adipokine-centred model does not capture. The resulting sarcopenic obesity phenotype—reduced muscle mass and function coexisting with excess adiposity—creates a state of dual metabolic vulnerability with compounded cardiometabolic and functional risk [12,15,33,38,39].

The EWGSOP2 diagnostic framework prioritises functional assessment: probable sarcopenia is identified by reduced handgrip strength or impaired chair stand performance; confirmed by demonstration of reduced skeletal muscle quantity or quality; and designated severe when strength, quantity/quality, and physical performance are all concurrently impaired. BIA provides a validated instrument for the quantity/quality dimension through skeletal muscle mass index (SMI = skeletal muscle mass/height^2^), derived from multi-frequency impedance measurements calibrated against DXA reference populations. Integration with handgrip dynamometry provides the composite assessment required for EWGSOP2 staging and for monitoring therapeutic responses in sarcopenic obesity [15,33].

### 5.5. BIA, Epigenetic Clocks, and the Dynamic Biological Age Concept

Epigenetic clocks based on DNA methylation at specific CpG sites—Horvath’s pan-tissue clock (353 CpG sites), Hannum’s blood clock, PhenoAge, and GrimAge—estimate biological age independently of chronological age and consistently reveal epigenetic age acceleration in obese subjects. Visceral fat area emerges as a stronger predictor of clock acceleration than BMI or total fat mass, confirming a specific ageing burden of the visceral compartment [12].

Circulating NAD^+^ level provides a complementary molecular longevity biomarker: its decline—driven by chronic NF-κB-dependent PARP activation and reduced NAMPT expression—directly connects the inflammatory state of visceral obesity to sirtuin suppression, mitochondrial dysfunction, and accelerated senescence. The convergence between BIA vector patterns and epigenetic clock acceleration in the same visceral obese subjects is mechanistically plausible: BIA provides the macroscopic cellular-level readout; the epigenetic clock provides the molecular validator. Integration of these two layers could generate a composite “dynamic biological age” metric for longevity assessment in outpatient settings, but this integration remains a research proposal rather than a validated clinical tool, and is identified in Section 7 as a high-priority target for prospective interventional study [35].

## 6. Therapeutic Strategies: Targeting the VAT-Inflammaging-BIA Axis

### 6.1. Anti-Inflammatory Nutrition and Caloric Modulation

Dietary intervention is the foundational strategy for VAT reduction and inflammaging control. The Mediterranean dietary pattern—high in vegetables, legumes, whole grains, olive oil, oily fish (EPA/DHA-rich), and polyphenol-rich foods, with restricted ultra-processed foods (NOVA group 4), refined sugars, saturated fats, and red meat—has the strongest evidence base for reducing VAT and systemic inflammatory markers, and its proposed active mechanisms extend beyond simple caloric effects to direct pharmacological-like modulation of inflammatory and metabolic pathways [40,41,42].

Ultra-processed foods warrant specific mechanistic attention beyond their macronutrient profile. Industrial emulsifiers directly disrupt the intestinal mucus layer and tight junction proteins; wheat amylase-trypsin inhibitors (ATIs) activate TLR4-mediated intestinal inflammation; processing-derived AGEs promote RAGE/NF-κB signalling. Large-scale epidemiological data confirm a dose–response relationship between UPF consumption and the risk of visceral obesity, metabolic syndrome, cardiovascular disease, and all-cause mortality [36,43,44].

Dietary fibre (target ≥ 25–30 g/day) and polyphenols promote intestinal butyrate production through SCFA-generating microbiota, directly suppressing NF-κB in colonic epithelium. Adequate protein distribution (1.2–1.6 g/kg/day in active adults, higher in sarcopenic individuals) is essential for preserving active cell mass. Caloric restriction of 20–30% below habitual intake activates the AMPK/NAD^+^/SIRT3-4 axis through mTORC1 suppression; time-restricted eating (TRE) protocols recapitulate these effects through circadian alignment, with randomised controlled trial evidence demonstrating significant reductions in VAT, fasting insulin, and hs-CRP at 8–12 weeks. It is worth noting, in this context, that population-level observations of an “obesity paradox”—improved survival associated with higher BMI in certain older or comorbid populations—complicate a simple message of unconditional weight and VAT reduction, and underscore why this review frames active cell mass preservation, and not weight loss alone, as the appropriate therapeutic target in older adults [35,41,42,45].

### 6.2. Exercise as a Mitochondrial and Anti-Senescent Intervention

Physical exercise is the most powerful non-pharmacological activator of the NAD^+^/sirtuin axis and mitochondrial biogenesis. Aerobic exercise activates AMPK, stimulates NAD^+^ synthesis through NAMPT upregulation, derepresses SIRT1 and SIRT3, and promotes PGC-1α-driven mitochondrial biogenesis. Exercise-induced mitophagy clears dysfunctional mitochondria that would otherwise contribute to mtROS overproduction and inflammasome activation. Both aerobic and resistance training reduce circulating IL-6, TNF-α, and hs-CRP; improve insulin sensitivity through GLUT4 upregulation; and reduce senescent cell burden in adipose tissue and skeletal muscle. Evidence-based recommendations target 150–300 min/week of moderate aerobic activity plus at least two resistance sessions weekly, with resistance training assuming particular importance given the sex-hormone-mediated component of sarcopenic obesity discussed in Section 5.4 [26,35,39,46].

### 6.3. Nutraceutical Support of the NAD^+^/Sirtuin and Redox Axes

Among nutraceutical agents with mechanistic relevance to the VAT-inflammaging axis: curcumin modulates NF-κB, NLRP3, and COX-2, reducing IL-6, TNF-α, and CRP; improved-bioavailability formulations are required. Resveratrol and quercetin activate SIRT1/AMPK pathways and induce Nrf2 target gene expression. Omega-3 long-chain polyunsaturated fatty acids (EPA/DHA) serve as biosynthetic precursors of specialised pro-resolving mediators (SPMs: resolvins, protectins, maresins) that actively promote inflammation resolution, and more broadly, polyunsaturated fatty acids modulate the fat mass-to-lean mass balance itself, a relevant consideration for sarcopenic obesity [13,40,47,48].

Mitochondrial-targeted agents include coenzyme Q10, pyrroloquinoline quinone (PQQ), and alpha-lipoic acid (restoring glutathione and mitochondrial redox balance). N-acetylcysteine (NAC) sustains the primary endogenous thiol-based antioxidant defence. For sarcopenic obesity, BCAA supplementation—particularly leucine, which directly activates mTORC1-mediated muscle protein synthesis—must be integrated with resistance training. Across this nutraceutical literature, effect sizes are frequently modest and trial durations short; head-to-head comparisons between agents, and confirmation in populations selected specifically for visceral obesity rather than general metabolic syndrome, remain a research gap [13,47,49].

**Figure 3:**
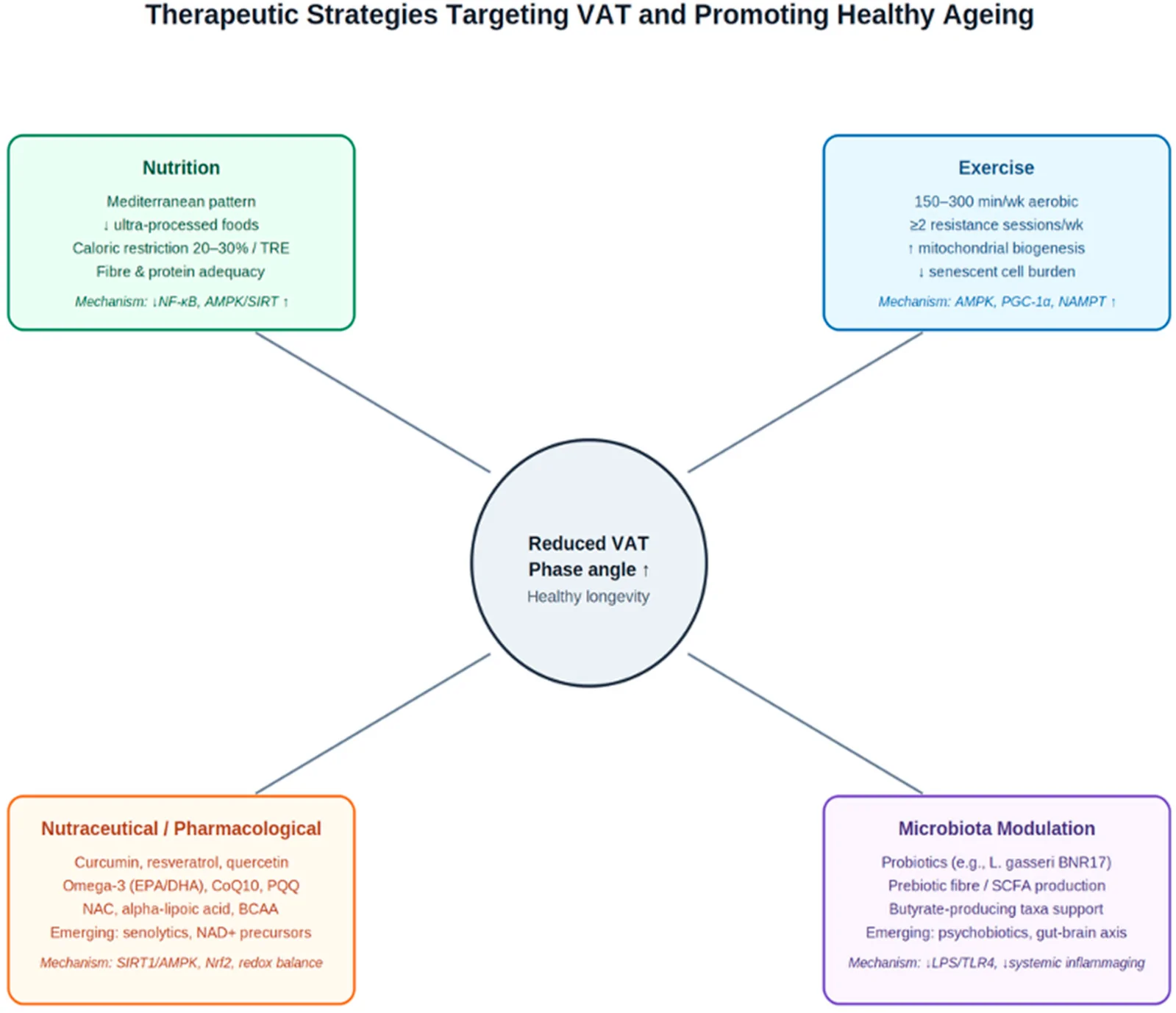
Therapeutic strategies targeting the VAT–inflammaging–BIA axis (see Section 6).

Microbiome-directed interventions represent a mechanistically coherent complement to anti-inflammatory nutrition. Lactobacillus gasseri BNR17 supplementation significantly reduced visceral fat area and waist circumference in a randomised, double-blind, placebo-controlled trial. Prebiotic supplementation (inulin, fructooligosaccharides, resistant starch) promotes SCFA-producing bacteria and butyrate availability, suppressing LPS-driven inflammaging. The emerging field of psychobiotics—targeting the gut–brain–immune axis—may additionally address the HPA axis dysregulation and circadian disruption that independently accelerate VAT accumulation and inflammaging, though this remains one of the least mature areas of the therapeutic literature reviewed here and is highlighted in Section 7 as a priority for adequately powered trials [36,46,50]. The full therapeutic framework is illustrated in Figure 3.

## 7. Discussion

This review has examined a specific and, we believe, clinically useful thesis: that visceral obesity and biological ageing operate through substantially overlapping molecular networks, converging at the cellular level in a shared BIA electrobiological signature. The chronic activation of NF-κB and NLRP3 by VAT-derived adipokines, the suppression of the NAD^+^/sirtuin axis by overnutrition, the accumulation of dysfunctional mitochondria through AMPK inhibition, the buildup of SASP-secreting senescent cells, and the progressive acceleration of epigenetic clocks are consistent with visceral obesity engaging the molecular mechanisms of ageing prematurely. This convergence, however, is best characterised as a well-supported mechanistic hypothesis rather than an established fact: as detailed in Section 3, Section 4 and Section 5, much of the supporting human evidence is cross-sectional and correlative, several links (stem cell exhaustion, intercellular communication) are less directly demonstrated than others (mitochondrial dysfunction, senescence), and the causal direction between specific molecular changes and clinical outcomes is not always established.

The diagnostic implications, while promising, should be stated with corresponding caution, distinguishing the disease-specific prognostic uses of phase angle that are genuinely established (Section 5.2) from its extension to visceral obesity and biological ageing, which remains exploratory (Section 5.3). The similarity between the BIA profile of visceral obese subjects and that of frail elderly individuals suggests that phase angle and the BIVA vector may provide a window into biological age that is partly independent of chronological age and BMI, accessible non-invasively in any clinical setting. The “metabolically obese, normal-weight” phenotype—subjects with excess VAT and impaired cellular health masked by normal BMI, as discussed in Section 3.5 alongside the broader methodological limitations of BMI-based screening—is plausibly a population for whom this diagnostic value is highest, though this specific application has not yet been validated in dedicated prospective cohorts. As emphasised throughout Section 3.5, no single assessment method—BMI, waist circumference, CT/MRI, DXA, or BIA—is universally superior; each occupies a distinct niche defined by accessibility, cost, and the specific clinical question being asked (Table 2, Figure 2).

Therapeutically, this convergence, if confirmed, would redefine treatment objectives. Weight reduction, while necessary, appears insufficient as a sole endpoint: evidence that remission of obesity does not fully restore mitochondrial homeostasis in visceral adipose tissue [34] indicates that cellular damage can persist beyond anthropometric normalisation. Therapeutic goals should therefore plausibly be expanded to include phase angle normalisation, active cell mass preservation, ICW/ECW balance restoration, and NAD^+^ repletion—while bearing in mind the obesity-paradox literature discussed in Section 6.1, which cautions against treating weight and VAT reduction as unconditionally beneficial across all age and comorbidity strata.

Knowledge gaps and priorities for future research. Several limitations must be acknowledged, and these define concrete priorities rather than generic caveats. First, as a narrative review, this work is susceptible to thematic selection bias and has not applied formal systematic-review methodology; a systematic review or scoping review of the BIA-longevity literature specifically would be a valuable complementary undertaking. Second, BIA-based predictive models for VFA and SMI are population-specific and require ethnic-group recalibration for non-European populations, a gap that limits generalisability of much of the cited evidence. Third, the claim that BIA phase angle is a macroscopic readout of epigenetic clock acceleration is supported by convergent but indirect evidence and has not yet been directly tested in longitudinal interventional studies with paired BIA and methylation clock measurements; such studies represent the single highest-priority research gap identified by this review. Fourth, the relative contribution of adipose tissue cellular heterogeneity and immunometabolic reprogramming (Section 3.1) to clinically measurable outcomes remains to be translated from single-cell and mechanistic studies into validated clinical biomarkers. Fifth, head-to-head, adequately powered trials comparing nutraceutical and microbiome-directed interventions (Section 6.3) specifically in populations selected for visceral obesity, rather than general metabolic syndrome, are lacking. Addressing these gaps would require coordinated prospective cohorts pairing BIA, imaging, epigenetic clocks, and inter-organ biomarkers over time—a substantially more resource-intensive undertaking than the cross-sectional and mechanistic evidence base that currently predominates.

## 8. Conclusions

Visceral obesity is among the most clinically prevalent candidate accelerators of biological ageing in the contemporary population. Through the sustained activation of the VAT-adipokine-NF-κB-inflammaging axis and the progressive suppression of the NAD^+^/sirtuin and AMPK/mTOR longevity axes, excess VAT engages multiple hallmarks of cellular ageing to varying degrees, contributing to a pathological continuum from metabolic syndrome through sarcopenic frailty, neurodegeneration, and oncogenesis.

BIA, and phase angle in particular, translates part of this molecular complexity into a single, non-invasive, clinically deployable measurement—one that should be understood as complementary to, rather than a replacement for, anthropometric and imaging-based assessment (Section 3.5). The inflammaging BIA signature of visceral obesity appears to be a macroscopic cellular expression of processes that epigenetic clocks and circulating NAD^+^ assays measure at the molecular level, though this convergence awaits direct longitudinal confirmation. Optimising phase angle, preserving active cell mass, and reducing VAT through evidence-based lifestyle, nutritional, and nutraceutical strategies constitute measurable and plausibly attainable objectives for the promotion of healthy longevity, contingent on the prospective, mechanistic, and comparative studies identified in Section 7 as this field’s principal priorities.

## Figures and Tables

**Table 1:** Principal BIA-derived parameters and their clinical significance in the assessment of visceral obesity and biological ageing.

Parameter	Reference Range	Clinical Significance
Phase angle (PhA)	5–8° (optimal 6–7°)	Primary candidate biomarker of cellular integrity and biological age; reduced in visceral obesity and frailty
ICW/ECW ratio	~2/1	Reflects intracellular vs. extracellular fluid balance; low ratio indicates sarcopenia or cellular senescence
ECW/TBW (AEC ratio)	0.360–0.400	Elevated values indicate shift toward extracellular fluid; marker of oedema, inflammaging, or lean mass loss
Active Cell Mass (ACM)	Sex/age-specific norms	Metabolically active tissue mass; candidate longevity parameter; reduced by VAT-driven catabolism
Skeletal muscle index (SMI)	≥7.0 kg/m^2^ (M), ≥5.5 (F)	EWGSOP2 diagnostic criterion for sarcopenia; estimated by BIA calibrated against DXA
Visceral fat area (VFA)	<100 cm^2^ (low risk)	Estimated from segmental BIA via population-specific predictive models; longitudinal monitoring of VAT reduction

**Table 2:** Comparison of methods for assessing visceral adiposity: clinical applicability, strengths, and limitations.

Method	Clinical Applicability	Strengths	Limitations
BMI	Universal screening, epidemiology	Simple, inexpensive, ubiquitous, large reference data sets	Cannot distinguish fat from lean mass or fat distribution; misses “metabolically obese, normal-weight” phenotype
Waist circumference	Routine clinical screening	Simple, low-cost, captures central adiposity	Technique- and observer-dependent; does not quantify VAT directly
Waist-to-hip ratio/Visceral Adiposity Index (VAI)	Cardiometabolic risk stratification	Improves on BMI/waist alone by incorporating fat distribution and, for VAI, lipid parameters	Still a surrogate index; validation across ethnic groups is incomplete
CT/MRI	Research reference standard; select high-risk patients	Direct, precise visceral fat area quantification	High cost, limited accessibility; radiation exposure (CT); impractical for routine or repeated use
DXA	Body composition assessment in clinical and research settings	Whole-body and regional fat/lean mass with modest radiation	Visceral fat estimates derived from equations, not direct visualisation; equipment cost
Bioelectrical impedance analysis (BIA)	Outpatient longitudinal monitoring; sarcopenic obesity assessment	Inexpensive, portable, radiation-free, repeatable; phase angle adds a functional/cellular dimension	Population-, ethnicity-, and equation-specific; only moderate agreement with imaging in some populations

## Data Availability

No new data were created or analyzed in this study. Data sharing is not applicable to this article.

## Abbreviations

The following abbreviations are used in this manuscript:

**VAT**	Visceral Adipose Tissue
**SAT**	Subcutaneous Adipose Tissue
**BIA**	Bioelectrical Impedance Analysis
**PhA**	Phase Angle
**ACM**	Active Cell Mass
**FFM**	Fat-Free Mass
**ICW**	Intracellular Water
**ECW**	Extracellular Water
**TBW**	Total Body Water
**VFA**	Visceral Fat Area
**SMI**	Skeletal Muscle Index
**SASP**	Senescence-Associated Secretory Phenotype
**BIVA**	Bioelectrical Impedance Vector Analysis
**EWGSOP2**	European Working Group on Sarcopenia in Older People (2nd revision)
**ROS**	Reactive Oxygen Species
**TNF-α**	Tumour Necrosis Factor-alpha
**IL-6**	Interleukin-6
**hs-CRP**	High-sensitivity C-Reactive Protein
**NAFLD**	Non-Alcoholic Fatty Liver Disease
**PCOS**	Polycystic Ovary Syndrome
**OSAS**	Obstructive Sleep Apnoea Syndrome
**AMPK**	AMP-activated Protein Kinase
**mTORC1**	Mechanistic Target of Rapamycin Complex 1
**PGC-1α**	Peroxisome Proliferator-Activated Receptor Gamma Coactivator 1-alpha
**NAD^+^**	Nicotinamide Adenine Dinucleotide (oxidised form)
**SIRT**	Sirtuin
**NF-κB**	Nuclear Factor kappa B
**NLRP3**	NOD-, LRR- and Pyrin Domain-containing Protein 3
**NAC**	N-Acetylcysteine
**BCAA**	Branched-Chain Amino Acid
**BMI**	Body Mass Index
**DXA**	Dual-Energy X-ray Absorptiometry
**DDR**	DNA Damage Response
**PARP**	Poly(ADP-ribose) Polymerase
**NAMPT**	Nicotinamide Phosphoribosyltransferase
**SCFA**	Short-Chain Fatty Acid
**SPM**	Specialised Pro-resolving Mediator
**TRE**	Time-Restricted Eating
**ATI**	Amylase-Trypsin Inhibitor
